# Heterochronic Shift in *Hox*-Mediated Activation of *Sonic hedgehog* Leads to Morphological Changes during Fin Development

**DOI:** 10.1371/journal.pone.0005121

**Published:** 2009-04-13

**Authors:** Koji Sakamoto, Koh Onimaru, Keijiro Munakata, Natsuno Suda, Mika Tamura, Haruki Ochi, Mikiko Tanaka

**Affiliations:** 1 Graduate School of Bioscience and Biotechnology, Tokyo Institute of Technology, Nagatsuta-cho, Midori-ku, Yokohama, Japan; 2 Institute of Neuroscience, University of Oregon, Eugene, Oregon, United States of America; Ecole Normale Supérieure de Lyon, France

## Abstract

We explored the molecular mechanisms of morphological transformations of vertebrate paired fin/limb evolution by comparative gene expression profiling and functional analyses. In this study, we focused on the temporal differences of the onset of *Sonic hedgehog* (*Shh*) expression in paired appendages among different vertebrates. In limb buds of chick and mouse, *Shh* expression is activated as soon as there is a morphological bud, concomitant with *Hoxd10* expression. In dogfish (*Scyliorhinus canicula*), however, we found that *Shh* was transcribed late in fin development, concomitant with *Hoxd13* expression. We utilized zebrafish as a model to determine whether quantitative changes in *hox* expression alter the timing of *shh* expression in pectoral fins of zebrafish embryos. We found that the temporal shift of Shh activity altered the size of endoskeletal elements in paired fins of zebrafish and dogfish. Thus, a threshold level of *hox* expression determines the onset of *shh* expression, and the subsequent heterochronic shift of Shh activity can affect the size of the fin endoskeleton. This process may have facilitated major morphological changes in paired appendages during vertebrate limb evolution.

## Introduction

There has been considerable debate regarding the fundamental mechanisms that direct morphological transformations from fins into limbs with respect to the expression patterns of 5′-located *Hox* genes and subsequent *Shh* expression [1], [2], [3]. It is generally accepted, however, that, the enlargement of the fin endoskeleton along the proximal-distal axis within the lineage of basal sarcopterygians (lobe-finned fishes) results from changes in the heterochronic folding of the apical fin fold [4]; other possibilities have scarcely been discussed. Here we have investigated the genetic basis of morphological transitions of the vertebrate fin endoskeleton primarily via comparative gene expression profiling and functional analyses, focusing especially on the temporal onset of *Shh* expression. Because two paired appendages are unique to gnathostomes—and cartilaginous fish occupy the earliest branch of the gnathostome lineage—the study of the cartilaginous dogfish may provide insight into how animals have acquired morphologically diverse paired appendages. Although the developmental mechanisms of such morphological changes are still under debate, the evolutionary acquisition of Shh function in growing paired appendages might have been a crucial step in implementing morphological innovations of paired appendages.

Patterning along the anterior-posterior axis of the limb is controlled by signalling from the posterior margin of the limb bud, the polarizing region discovered by Saunders and Gasseling [5]. Grafted tissue from the polarizing region of a chick limb bud to the anterior margin of another chick limb bud resulted in remarkable mirror-image symmetry of digits. Several subsequent studies showed that this polarizing activity involves a dose-dependent response because the identity of the additional digits that form depends on the number of grafted cells from the polarizing region [6], [7]. *Sonic hedgehog* (*Shh*), which encodes a secreted factor, was later found to be expressed precisely in those cells identified as the polarizing regions in the limb buds of chick and mouse [8], [9] and also in zebrafish fin buds [10]. Application of *Shh*-expressing cells or an Shh-soaked bead into the anterior margin of chick limb buds induced the same type of dose-dependent mirror-image digit patterns as a graft tissue from the polarizing region [8], [11]. More recently, it was shown that the length of time that cells are exposed to Shh, in addition to the Shh dose, is crucial for the patterning of the digital plate [12], [13]. Subsequent experiments demonstrated that the longer the limb bud cells are exposed to Shh, the more posterior digits are formed [13]. Furthermore, recent studies demonstrated that Shh can regulate not only digit specificity but also cell proliferation in limb buds of chick and mouse embryos [14], [15]. Similarly, a requirement for shh acitivity in cell proliferation in the zebrafish pectoral fin bud has also been suggested [16]. These results raise the possibility that the duration of exposure to Shh activity may have been critical for the morphological evolution of paired appendages.

To investigate the possibility that the duration of exposure to Shh activity may have been critical for the morphological evolution of paired appendages, we analyzed fin development in embryos of the cartilaginous dogfish *Scyliorhinus canicula*. In limb buds of chick and mouse, *Shh* expression is activated as soon as there is a morphological bud, whereas in *S. canicula* fin buds, consistent with reported data in other cartilaginous fishes [17], *Shh* is transcribed late in fin development. Several molecular triggers that activate *Shh* expression have been proposed, including Hand2 and Fibroblast growth factor (Fgf) [18], [19]. In pectoral fins of *S. canicula*, *Hand2* transcripts localize posteriorly at a much earlier stage than *Shh* transcripts, and it is therefore unlikely that *Hand2* correlates directly with the late onset of *Shh* transcription [20]. In vertebrate limb buds, Fgfs are secreted from the apical ectodermal ridge that rims the distal edge of the buds, and these Fgfs play pivotal roles in limb bud initiation and outgrowth, at least in part by inducing and maintaining the expression of *Shh* in the underlying mesenchyme [19], [21], [22], [23], [24]. *Hoxa* and *Hoxd* have also been demonstrated to drive *Shh* expression in mouse limb buds [2], [3], [25]. Furthermore, recent experiments have shown that Hox proteins bind to a conserved regulatory region of *Shh*, thereby promoting *Shh* expression within developing mouse limb buds [26]. In our current study, we show that a threshold level of *hox* expression is essential for the onset of *shh* expression and that the subsequent heterochronic shift of Shh activity leads to changes in the size of pectoral fins. These results imply that a quantitative change in *hox* expression could have involved a heterochronic shift of *shh* expression and subsequent morphological changes of endoskeleton during limb evolution.

## Materials and Methods

### Animals

*S. canicula* eggs were incubated at 12∼16°C in sea water and staged according to Ballard et al. (1993). The gross duration of incubation described in Ballard et al. (1993) was as follows: stage 27 (42–46 days), stage 29 (49–53 days), stage 32 (75–125 days). Because duration of stage 32 is long, we subdivided stage 32 into “early stage 32” (75–100 days) and “late stage 32” (101–125 days). Wild-type (TL strain and AB/Tübingen strain) zebrafish (*Danio rerio*) were maintained at 28.5°C and staged using standard morphological criteria [27].

### Identification of *S. canicula* gene homologs

We identified fragments of *S. canicula* (*Sc*) *Fgf8* (296 bp), *Meis1* (357 bp), *Hoxa11* (357 bp), *Hoxa13* (389 bp), *Hoxd11* (534 bp), *Hoxd13* (296 bp), *Pbx2* (653 bp), *Ptc2* (1157 bp) and *GAPDH* (230 bp) from cDNA pools prepared from stage 24–30 embryos using degenerate primers. The degenerate primers were designed to anneal to coding regions containing the following amino acid sequences: *ScFgf8*, TYQLYSRT and VHFMKRL; *ScMeis1*, CDNFCHR and GIFPKVA; *ScHoxa11*, QVQPVRE and AATSSS; *ScHoxa13*, AYTSSEV and PMESYQP; *ScHoxd11*, CQMTFPYS and PYTKYQIR; *ScHoxd13*, PVEKYMDV and IWFQNRRV; *ScPbx2*, QQIMTIT and PYPSEEA; *ScPtc2*, IHAFSTT and QFKYFSFYNF; *ScGAPDH*, ASCTTN and VIPELN. *S. canicula ScHoxd10* (785 bp) and *ScHoxd12* cDNAs (316 bp) were amplified by PCR using the following primers which hybridized to the indicated published sequence: *ScHoxd10*, GenBank accession number DQ659105, 5′-GGGAACATACGGAATGCAGACC-3′ and 5′-GTAAGAGCGTGAATCTGACCG-3′; *ScHoxd12*, GenBank accession number DQ659106, 5′-CCCTTCTATTTCGCCAACCTG-3′ and 5′-CCCAAGTGATACCAGCATCC-3′. The nucleotide sequences of the *ScFgf8*, *ScHoxd13*, *ScMeis1*, *ScHoxd11*, *ScHoxa11*, *ScHoxa13*, *ScPbx2*, *ScPtc2* and *ScGAPDH* cDNAs were deposited in the GenBank database under the accession numbers: DQ647321–DQ647323, DQ854846, EU005549–EU005551, EU814484 and EU826015, respectively.

### Whole-mount in situ hybridization and immunohistochemistry

*S. canicula* embryos were removed from their egg casings and dissected from the yolk mass. Whole-mount *in situ* hybridization of *S. canicula* and immunostaining of *S. canicula* embryos were carried out as described [20]. Whole-mount *in situ* hybridization of zebrafish was performed as described [28]. Probes for zebrafish *hoxd10a*, *hoxd11a* and *hoxd13a* were amplified by reverse transcription-polymerase chain reaction (RT-PCR) using primers derived from published sequences (www.ensembl.org). For whole-mount immunostaining, embryos were prepared as described [29]. The monoclonal antibody against human Fgf4 (R & D Systems) was used at a 1∶300 dilution.

### Microinjection

For mRNA injection, the full-length cDNAs encoding *hoxa13a*, *hoxd10a*, *hoxd13a*, *hoxd4* and *pbx2* were individually cloned into the pCS2+ vector and the corresponding mRNAs were synthesized using the MEGAscript kit (Ambion). The mRNAs were dissolved in endotoxin-free H_2_O to a final concentration of 20 mg/ml. Morpholino antisense oligonucleotides (MOs) were obtained from Gene Tools, Inc. The following *hoxd10a* and *hoxd13a* MOs targeted the boundary between exon 1 and intron 1 of each respective gene (Gene Tools, Inc.): MO-*hoxd10a*, CCGTTTATTGTACCCACCTTTGCCT; MO-*hoxd13a*, CAGAGCTGAGGTCTTACCTGTTAAT. The *pbx2* MO was used as described [30]. The standard control MO obtained from Gene Tools, Inc. was used as an injection control. MOs were dissolved in sterile H_2_O at concentrations of 1, 2.5 or 5 mg/ml and phenol red was added to the solution. Approximately 1 nl of mRNA or MO was injected at the one-cell stage using a microinjector (IM30, Narishige).

To test the efficiency of the *hoxd10a*-MO and the *hoxd13a*-MO, RT-PCR was performed using total RNA from 30 embryos at 24 hpf to detect spliced and unspliced *hoxd10a* or *hoxd13a* mRNAs. The following PCR primers for *hoxd10a* and *hoxd13a* were used for amplification: *hoxd10a*, 5′-TGTCCACCTGCACATTTTCAC-3′ and 5′-CTTGTCTGTCAGTCAGGTTGACGC-3′; *hoxd13a*, 5′-GAGATCTTAGACATGAGACTTG-3′ and 5′-CCTCTTTGAATTCGAGATTCTC-3′. Amplification of *eif4a* transcripts was used as a control [31].

### Semi-quantitative and quantitative expression analysis

Lateral plate mesoderm overlying the yolk of zebrafish embryos and pectoral fin buds of dogfish embryos were isolated by dissection. Total RNA was extracted from dissected embryos using the RNeasy Mini kit (Qiagen). To remove genomic DNA, each RNA sample was treated with RNase-free DNase (Qiagen). The RNA was used as a template for synthesizing cDNA using AMV Reverse Transcriptase (Promega). The following PCR primers for *ScFgf8* were used for amplification: 5′-AGATTAACGCAAAGGCGGAGG-3′ and 5′-GAATCAATGCTACTGCTGAAG-3′. For semi-quantitative RT-PCR, spliced, functional *hoxd10a*,*hoxd11a* and *ScShh* transcripts were amplified with the following primers: *hoxd10a*, 5′-CCAAAGTCAGCACGCTGGAG-3′ and 5′-CTCCCGAGTCAGATACATGTTG-3′; *hoxd11a*, 5′-ACACCGTGGAGGAGGAATCC-3′ and 5′-CGTTCAAGTTCTCGGATCTGG-3′; *ScShh*, 5′-CTGACAGGCTGATGACACAG-3′ and 5′-ATCCCGTACTTGGTTCGGTC-3′. To determine relative transcript levels of functional *hoxd10a*,*hoxd11a*, and *ScShh* RT-PCR products were subjected to agarose gel electrophoresis, soaked in a 1 µg/ml ethidium bromide solution, and the intensity of each band was measured using the ImageJ program (National Institute of Health, Bethesda, MD). For quantitative real-time RT-PCR, we used the 7300 real-time PCR System (Applied Biosystems) with SYBR Green I. *hoxd11a*, *shh*, *ScHoxd10*, *ScHoxd11*, *ScHoxd12* and *ScHoxd13* transcripts were amplified with the following primers: *hoxd11a*, 5′- CCGTTTCAACCTGCGATGAAG -3′ and 5′- CGTTCAAGTTCTCGGATCTGG -3′; *shh*, 5′-TTGACTGGGTCTATTACGAGTCC-3′ and 5′-GGTTCAGGTCCTTCACGGCCTTC -3′; *ScHoxd10*, 5′- GAACTATCGGACAATGAGAC -3′ and 5′- CGGTCAGATTCACGCTCTTAC -3′; *ScHoxd11*, 5′- TCGGACACCTCTAACTATGAAC -3′ and 5′- ACACTGTTACCGGAGGACTC -3′; *ScHoxd12*, 5′- CCCTTCTATTTCGCCAACCTG -3′ and 5′- TGATGGAGACTGAGTTGCTG -3′; *ScHoxd13*, 5′- ACTGACGAGGTGTCATCCAG -3′ and 5′- TGCATCGCAGGTTAGTGGATAG -3′.

The relative expression level of each gene was normalized to *gapdh* expression [32] for zebrafish and *ScGAPDH* expression for dogfish embryos. Each standard deviation was calculated using data from three independent experiments.

### Cyclopamine and SAG treatment

To investigate the effect of hedgehog (hh) signaling on pectoral fin buds, zebrafish embryos were treated from 23 hpf to 27 or 57 hpf with either 0.6% (v/v) ethanol in fish water (vehicle) [28] or with 60 µM cyclopamine (Biomol), a hh signaling antagonist, dissolved in vehicle. Incubation with cyclopaminewas terminated by washing in fish water, and embryos were incubated until fixation. To examine the effect of hh signaling on adaxial cells, zebrafish embryos were treated from the 1-cell-stage to the 8-somite-stage with either 1.0% (v/v) ethanol in fish water (vehicle) [28], 100 µM SAG (Alexis), a hh signaling agonist, or 100 µM cyclopamine in vehicle.

Dogfish embryos were treated for 4 days from stage 28 with cyclopamine or 6 days from stage 30 with SAG. Briefly, 50 µl of 10 mM cyclopamine dissolved in ethanol or 25 µl of 100 µM SAG dissolved in ethanol was injected into the dogfish egg case, which then was reared in seawater. For SAG treatment, 25 µl of 100 µM SAG was added 3 days after the first day of treatment. Control embryos were reared in seawater. Incubation with cyclopamine or with SAG was terminated by washing in seawater several times, and embryos were reared in seawater until fixation.

### Cartilage staining

Cartilage staining was conducted as described [33].

## Results

### *Shh* is transcribed late in *S. canicula* development, concomitant with *Hoxd13* expression

**Figure 1:**
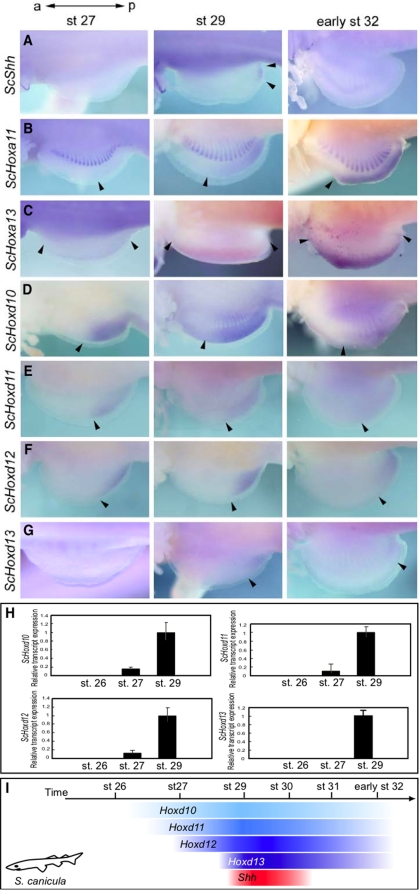
*Shh* expression commences late in *S. canicula* (*Sc*) fin development, concomitant with *Hoxd13* expression. (A–G) Pectoral fin buds. Anterior is to the left. (A) *ScShh* expression at stages 27, 29 and early stage 32. Transcripts were present in the posterior region (arrowheads) at stage 29 but absent at stages 27 and early stage 32. (B, C) Expression of *ScHoxa11* (B) and *ScHoxa13* (C). *ScHoxa11* transcripts were first detected in the posterior region and in the muscle buds. By early stage 32, transcripts were restricted to the posterior-distal region. *ScHoxa13* transcripts were restricted to the distal part of the fin buds throughout fin development. Arrowheads indicate limits of *ScHoxa* expression. (D–G) Expression of *ScHoxd10* (D), *ScHoxd11* (E), *ScHoxd12* (F) and *ScHoxd13* (G). The *ScHoxd* genes were expressed collinearly at early stages. *ScHoxd10–d12* transcripts were apparent at stage 27, whereas *ScHoxd13* transcripts were first observed in the posterior mesenchyme at stage 29. Arrowheads indicate the anterior limits of *ScHoxd* expression. (H) Quantitative PCR analysis to determine the expression levels of *ScHoxd10–13* in the pectoral fins of stage 26, 27 and 29 dogfish embryos. Relative expression was normalized against *ScGAPDH* transcripts. Note that levels of *ScHoxd10–13* transcript expression increased at stage 29. Expression of *ScHoxd10–d13* in stage 26 pectoral fins, or expression of *ScHoxd13* in stage 27 pectoral fins, was not detectable. (I) Schematic representation of temporal *Hoxd* expression and *Shh* expression during pectoral fin development in *S. canicula*. *Shh* was expressed concomitantly with *Hoxd13*.

The evolutionary acquisition of *Shh* function into growing paired appendages might have been crucial in implementing the morphological evolution of tetrapod appendages. We previously reported that *Shh* expression could not be detected in the fin buds of dogfish (*Scyliorhinus canicula*) embryos at stage 27 [20] and further studies have confirmed this finding (Fig. 1A). In addition, however, when we examined fin buds at much later stage 29, we detected posterior *Shh* expression (Fig. 1A). By early stage 32, *Shh* expression became downregulated in fin buds (Fig. 1A), as confirmed by RT-PCR analysis (Fig. S1)[20]. In contrast, *Shh* expression in chick and mouse is activated as soon as there is a morphological bud and persists at least until the distal region that will give rise to digits is produced [8]. This suggests that temporal shifts in the *Shh* expression during vertebrate limb evolution might have led to major morphological innovations and diversification in paired appendages. To explore this possibility further, we investigated several genetic components that may have contributed to acquisition of *Shh* expression in fins at this late stage of development in dogfish. We first examined whether Fgf signalling in *S. canicula* fins is reduced and/or delayed, leading to a delay in *Shh* expression in fin buds. Although the distal edge of *S. canicula* fin buds has an ectodermal structure called the apical fin fold that is similar to the apical ridge of limb buds of higher vertebrates, it is not known whether the apical fin fold produces Fgf. It is possible that Fgf is not produced at a time that would influence *Shh* expression. Therefore we isolated cDNA fragments of *Fgf8* from *S. canicula* embryos and examined their expression patterns at stages 27–32 (Fig. S1B–D). *In situ* hybridization experiments showed that *Fgf8* was expressed in the developing gill filaments and nasal pits of stage 27 *S. canicula* embryos (Fig. S1B). In contrast, *Fgf8* transcripts could not be detected in the apical fin folds at any stage examined (Fig. S1C–E). We also investigated production of Fgfs using an antibody against Fgf4. We found that anti-Fgf4 antibody-positive cells were distributed in the apical ectodermal fold at stage 27 (Fig. S1F). Wnt signaling induces *Fgf* expression via a *β-catenin*-dependent pathway in limb bud–forming regions in vertebrates. To test the probe efficacy in the apical fin fold of *S. canicula* fins, we isolated *β-catenin* cDNA fragments and examined their expression pattern. In *S. canicula* fins at stages 27 (not shown) to 32 (Fig. S1G), abundant *β-catenin* transcripts were observed, including in the apical fin fold (arrows in Fig. S1G), demonstrating probe efficacy. These results indicated that signaling by Fgfs occurs at early fin bud stages in *S. canicula* and may be involved in fin patterning and outgrowth. We therefore concluded that the late onset of *Shh* transcription in fin buds is not due to a delay in *Fgf* expression during the early bud stages.

The *Hox* genes have recently been shown to regulate *Shh* transcription in developing mouse limb buds. In higher vertebrates, ectopic *Hox* expression leads to *Shh* transcription, whereas functional ablation of *Hox* genes leads to distal limb truncations caused by the absence of *Shh* expression [2]. To investigate whether the late onset of *Shh* transcription in fin buds of *S. canicula* is regulated by *Hox* genes, we isolated cDNA fragments of the 5′-located *Hoxa* and *Hoxd* genes, such as *Hoxa11*, *Hoxa13*, *Hoxd10*, *Hoxd11*, *Hoxd12* and *Hoxd13* from *S. canicula* and examined their expression patterns. Very weak hybridization signal was seen for *Hoxa11* in the posterior fin buds and muscle buds at stage 27, but this signal intensified in later stages (Fig. 1B). *Hoxa13* expression appeared at stage 27 in the distal region and persisted in the same region at least until early stage 32 (Fig. 1C). Thus, expression of 5′ -located *Hoxa* genes in the developing pectoral fins in *S. canicula* was greater at stage 29 than at stage 27 and remained nested and overlapping throughout development in a manner remarkably similar to that seen in zebrafish [1] and *Polydon spathula* [34].

Collinear expression of *Hoxd* genes was also observed in the pectoral fins, in accordance with previous results [35]. Hybridization signals for *Hoxd10–12* were seen in the posterior region of the pectoral fins in a nested manner at stage 27 (Fig. 1D–F), whereas no transcripts of the 5′-most *Hoxd* gene, *Hoxd13*, were detected in pectoral fins of stage 27 embryos (Fig. 1G). By stage 29, when *Shh* expression is turned on, *Hoxd10–12* expression had increased (Fig. 1D–F), and *Hoxd13* expression appeared in the posterior part of the pectoral fin buds (Fig. 1G). At early stage 32, *Hoxd10* expression persisted in the posterior fins, but expression of *Hoxd11–13* had decreased (Fig. 1D–G). Thus, *Shh* expression was transcribed at stage 29 concomitantly with *Hoxd13* expression in pectoral fins of *S. canicula* embryos (Fig. 1I). To quantify the expression levels of *S. canicula Hoxd10–13* (*ScHoxd10–13*) in pectoral fin buds of embryos, we performed quantitative real-time PCR using total RNA from pectoral fin buds at stages 26, 27 and 29 (Fig. 1H). *ScHoxd10–13* mRNA levels in pectoral fin buds had dramatically increased by stage 29 (Fig. 1H). These results suggested that the temporal expression of *Hox* in the pectoral fins may correlate with the late onset of *Shh* transcription in *S. canicula* embryos.

### The level of *hox* transcripts is critical for the onset of *shh* expression in pectoral fin primordia of zebrafish embryos

**Figure 2:**
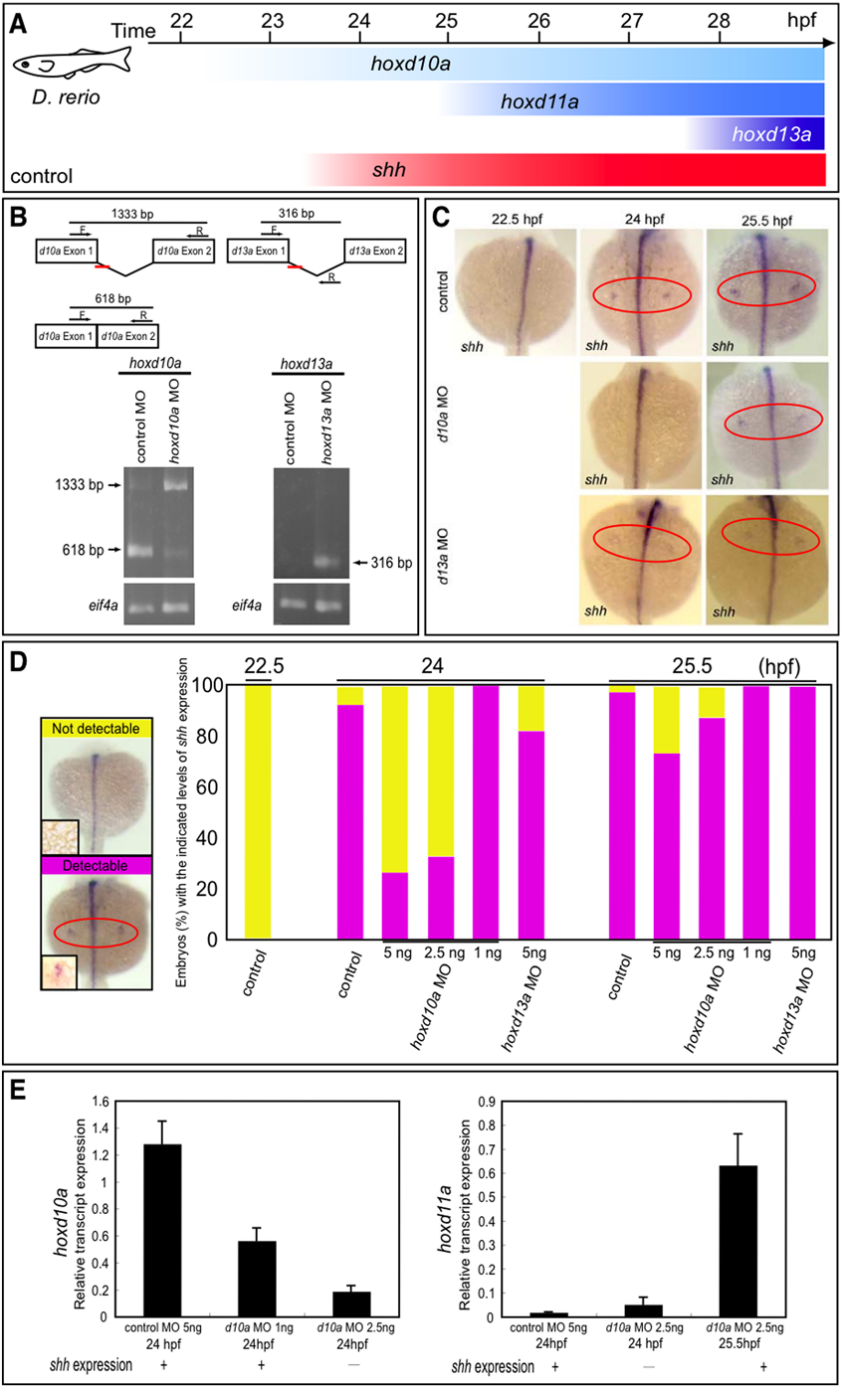
Timing of *shh* expression in zebrafish embryo fin primordia depends on *hox* transcript accumulation. (A) Schematic representation of temporal *hox* and *shh* expression in the pectoral fin primordia of zebrafish embryos. *shh* was expressed at 24 hpf concomitantly with *hoxd10a* expression. (B) RT-PCR analysis to determine the efficiency of the *hoxd10a* or *hoxd13a* splice-blocking morpholino (MO). In the schematics, arrows represent forward (F) and reverse (R) primers, and the short red bars represent the *hoxd10a* MO and *hoxd13a* MO. Lower panel, analysis of RT-PCR products by agarose gel electrophoresis. Products of 618 bp and 1333 bp represent spliced and unspliced *hoxd10a* mRNA, respectively. The 316-bp RT-PCR product represents spliced *hoxd13a* mRNA. Amplification of *eif4a* cDNA was used as a control. (C) Whole-mount *in situ* hybridization to detect *shh* expression in the pectoral fin primordia of *D. rerio* embryos injected with 5 ng control MO (top panels), 5 ng *hoxd10a* MO (middle panels) or 5 ng *hoxd13a* MO (bottom panels) at the indicated hpf. Red ovals highlight the pectoral fin primordia. Note that *shh* expression was first observed at 24 hpf in the fin primordia of embryos injected with control (top) or *hoxd13a* MO (bottom), whereas *shh* transcripts became detectable at 25.5 hpf in the primordia of most embryos injected with *hoxd10a* MO (middle). (D) Percentages of embryos with detectable or undetectable levels of *shh* expression observed at 22.5, 24, and 25.5 hpf following injection of control MO, *hoxd10a* MO or *hoxd13a* MO (see also Figure S4). A representative image depicting the detectable or undetectable levels of *shh* expression in the pectoral fin primordia is shown at the left. Insets show high magnification views of pectoral fin primordia. (E) Semi-quantitative RT-PCR analysis to determine the expression levels of 5′ *hoxd* when *shh* is transcribed in pectoral fin buds. The relative levels of *hoxd10a* and *hoxd11a* transcripts in the lateral plate mesoderm of morphants were quantified. Relative expression was normalized against *gapdh* transcripts.

**Figure 3:**
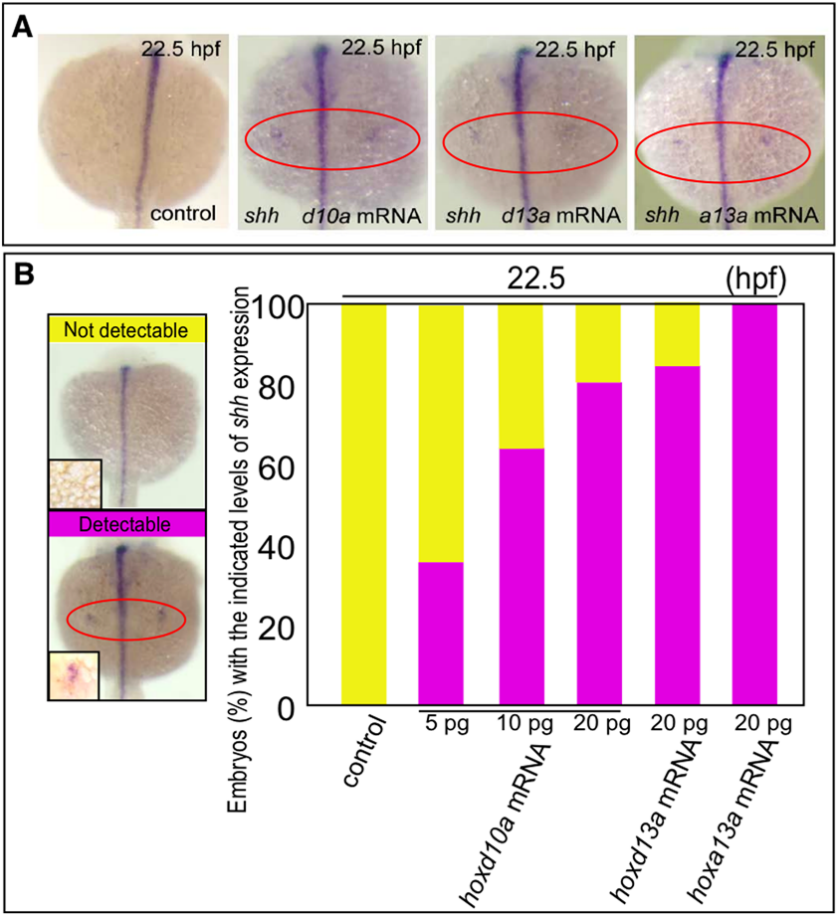
*hox* transcript accumulation is critical for the onset of *shh* expression in fin development. (A) Expression of *shh* in pectoral fin primordia of *D. rerio* embryos injected with 5 ng control MO, 20 pg *hoxd10a* mRNA, 20 pg *hoxd13a* mRNA or 20 pg *hoxa13a* mRNA at 22.5 hpf. Red ovals highlight the pectoral fin primordia. Note that transcripts of *shh* became detectable at 22.5 hpf in the fin primordia of embryos injected with *hoxd10a*, *hoxd13a* or *hoxa13a* mRNA. (B) The percentage of embryos with the indicated level of *shh* expression at 22.5 hpf following injection of control MO, *hoxd10a* mRNA, *hoxd13a* mRNA or *hoxa13a* mRNA is shown in the bottom panel (see also Figure S4). A representative image depicting the detectable or undetectable levels of *shh* expression in the pectoral fin primordia is shown at the left.

In the dogfish *S. canicula* pectoral fins, *Shh*, which is transcribed at a late stage in fin development, was expressed at the same time as *Hoxd13* (Fig. 1I). In contrast, *shh* expression in zebrafish occurred at 24 hours post-fertilization (hpf) and was concomitant with *hoxd10a* expression in pectoral fin primordia (Figs. 2A and C, Fig. S2). To address whether expression of the 5′-*hox* genes could shift the onset of *shh* transcription in pectoral fin primordia, we manipulated the expression levels of specific *hox* transcripts in the zebrafish model system (Figs. 2 and 3).

We used an antisense morpholino oligonucleotide (MO) to change the levels of *hoxd10a* or *hoxd13a* transcripts. The MOs were designed to inhibit splicing of *hoxd10a* or *hoxd13a* pre-mRNA, leading to the knockdown of *hoxd10a* or *hoxd13a* function. Unspliced *hoxd10a* transcripts were detectable by RT-PCR in embryos injected with 7.5 ng of the *hoxd10a* MO (1333-bp band in Fig. 2B, lower panel), whereas in embryos injected with the control MO, spliced *hoxd10a* mRNAs were detected (618-bp band in Fig. 2B, lower panel). We also detected unspliced *hoxd13a* transcripts in embryos injected with 7.5 ng of the *hoxd13a* MO, (316-bp band in Fig. 2B, lower panel), whereas no band was detected in embryos injected with 5 ng of the control MO (Fig. 2B, lower panel). These results demonstrated that the MOs targeting *hoxd10a* and *hoxd13a* efficiently blocked production of the mature *hoxd10a* and *hoxd13a* spliced transcripts.

We then examined the pectoral fins of *hoxd10a* or *hoxd13a* zebrafish morphants with those of control morphants at 24 and 25.5 hpf. Expression of *shh* was first observed in pectoral fin primordia of 24 hpf embryos injected with 5 ng control MO (91.2% of morphants, n = 34, Figs. 2C and D, Fig. S4). When 5 ng of *hoxd10a* MO was used, however, *shh* expression was initiated in only 28.1% of 24 hpf embryos (n = 32); by 25.5 hpf, *shh* was expressedin 72.7% of morphants (n = 33, Figs. 2C and D, Fig. S4). This delay in the onset of *shh* expression was also observed in 70.0% of embryos injected with 2.5 ng of *hoxd10a* MO (n = 30, Fig. 2D, Fig. S4). However, injection of a lower concentration of *hoxd10a* MO (1 ng) did not cause a delay in onset of *shh* expression in any morphants (n = 30, Fig. 2D, Fig. S4). In zebrafish, *hoxd13a* expression appeared in pectoral fin primordia at a much later stage (28 hpf, Fig. S2) than *shh* (24 hpf, Figs 2C). When we injected 5 ng *hoxd13a* MO into eggs, *shh* expression was observed in pectoral fin primordia of 24 hpf morphants (81.8%, n = 22, Figs. 2C and D, Fig. S4), similar to that for embryos injected with 5 ng control MO at 24 hpf (91.4%, n = 34, Figs. 2C and D, Fig. S4). Semi-quantitative RT-PCR showed that injection of increasing amounts of *hoxd10a* MO efficiently reduced the amount of spliced, functional *hoxd10a* transcripts in a dose-dependent manner from the lateral plate mesoderm of zebrafish morphants at 24 hpf (Fig. 2E). Transcription of *shh* also was first observed at 24 hpf in the pectoral fin primordia of embryos injected with 1 ng of *hoxd10a* MO (Fig. 2D), although the amount of spliced *hoxd10a* transcripts was reduced to 50% of that of control embryos (Fig. 2E). In contrast, *shh* expression was not observed in the pectoral fin primordia of zebrafish embryos injected with 2.5 ng *hoxd10a* MO (Fig. 2D), in which the amount of spliced *hoxd10a* transcripts was reduced to 15% of that of control embryos (Fig. 2E). Transcripts of functional *hoxd11a* were barely detectable in pectoral fin primordia of zebrafish embryos injected with either control MO or 2.5 ng *hoxd10a* MO. Expression of *shh* could be detected by *in situ* hybridization (Fig. 2C) by 25.5 hpf, when *hoxd11a* expression was detected in *hoxd10a* morphants (Fig. 2E), although the amount of functional *hoxd10a* transcripts was still effectively reduced. Results from the real-time quantitative RT-PCR analyses confirmed these observations (Fig. S3).

**Figure 4:**
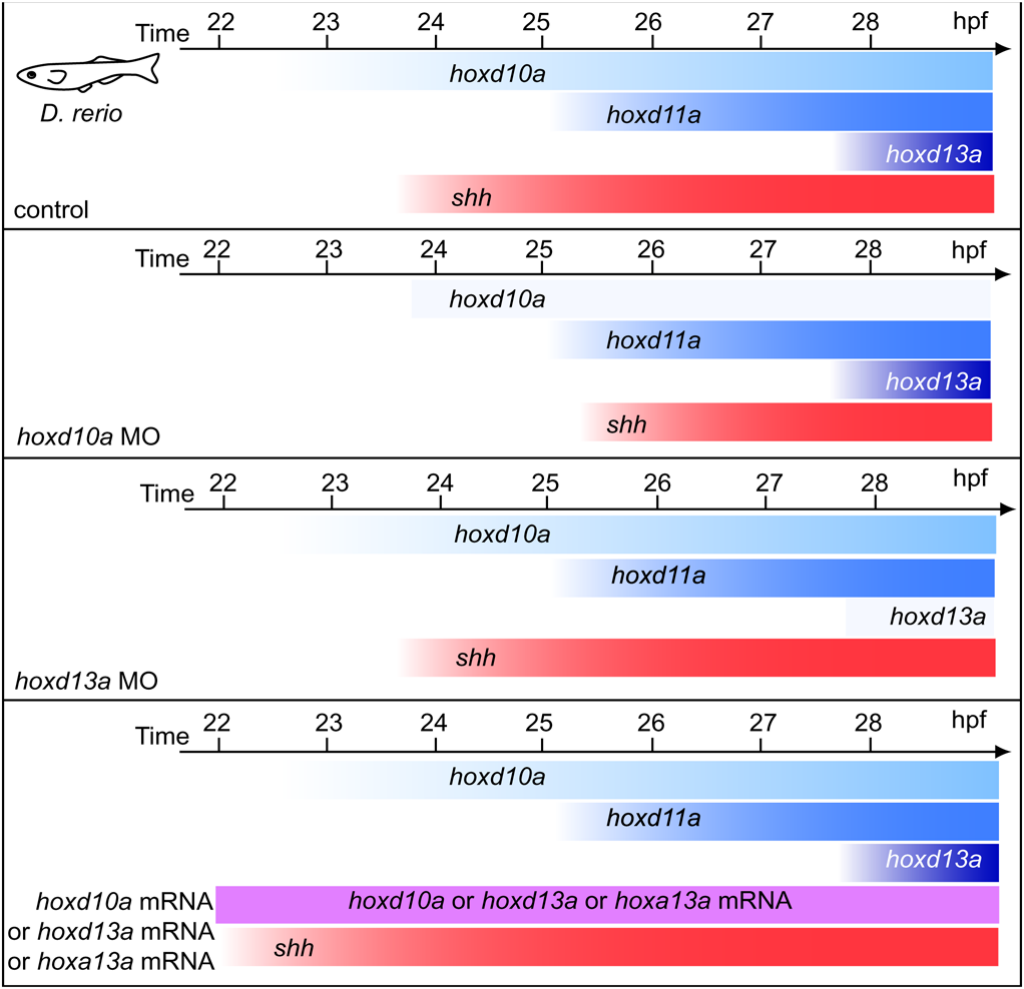
Schematic representation of temporal *hox* and *shh* expression in pectoral fin primordia of zebrafish embryos. Expression of *shh* was observed at 24 hpf and was concomitant with *hoxd10a* expression. The onset of *shh* expression in *hoxd10a* morphants was concomitant with the onset of *hoxd11a* expression, whereas *shh* expression was not delayed in *hoxd13a* morphants. In embryos injected with *hoxd10a*, *hoxd13a* or *hoxa13a* mRNA, *shh* expression was observed at 22.5 hpf.

Because the onset of *shh* expression in *hoxd10a* morphants coincided with the onset of *hoxd11a* expression (Fig. 2C and 4), it is possible that *shh* is transcribed only when a certain threshold level of accumulated *hox* is present in zebrafish pectoral fin primordia. To test this hypothesis, we injected *hoxd10a* mRNA or *hoxd13a* mRNA into embryos and investigated whether excess amounts of *hoxd* mRNA could accelerate the timing of onset of *shh* expression in pectoral fin primordia. Although control embryos did not express *shh* in pectoral fin primordia at 22.5 hpf (0%, n = 32, Fig. 3A, Fig. S4), 88% of embryos injected with 20 pg *hoxd10a* mRNA expressed *shh* in pectoral fin primordia at 22.5 hpf (n = 25, Fig. 3, Fig. S4). In embryos injected with *hoxd10a* mRNA, the onset of *shh* expression was accelerated in a dose-dependent manner (Fig. 3B, Fig. S4). These observations were confirmed by real-time quantitative RT-PCR analyses (Fig. S3D). Likewise, *shh* transcripts appeared at 22.5 hpf in 82.6% of embryos injected with 20 pg *hoxd13a* mRNA (n = 23, Fig. 3, Fig. S4). Thus, expression levels of *hoxd* are crucial for the timing of *shh* expression in zebrafish fin primordia. In mouse limb buds, *Hoxa* genes, as well as *Hoxd* genes, are involved in regulation of *Shh* expression [3]. We therefore investigated whether the onset of *shh* expression in fin primordia could also be triggered by a threshold level of *hoxa*. At 22.5 hpf, *shh* expression was seen in pectoral fin primordia in 100% of zebrafish embryos injected with 20 pg *hoxa13a* mRNA (n = 27, Fig. 3, Fig. S4). Thus, expressing a threshold level of *hoxa* could also trigger *shh* expression in pectoral fin primordia (Fig. 4). Our results indicate that specific threshold levels of *hox* gene products likely trigger the heterochronic shift of *shh* expression in pectoral fin primordia.

### Temporal shift of Shh activity leads to morphological changes in endoskeletal elements of pectoral fins in zebrafish and dogfish

**Figure 5:**
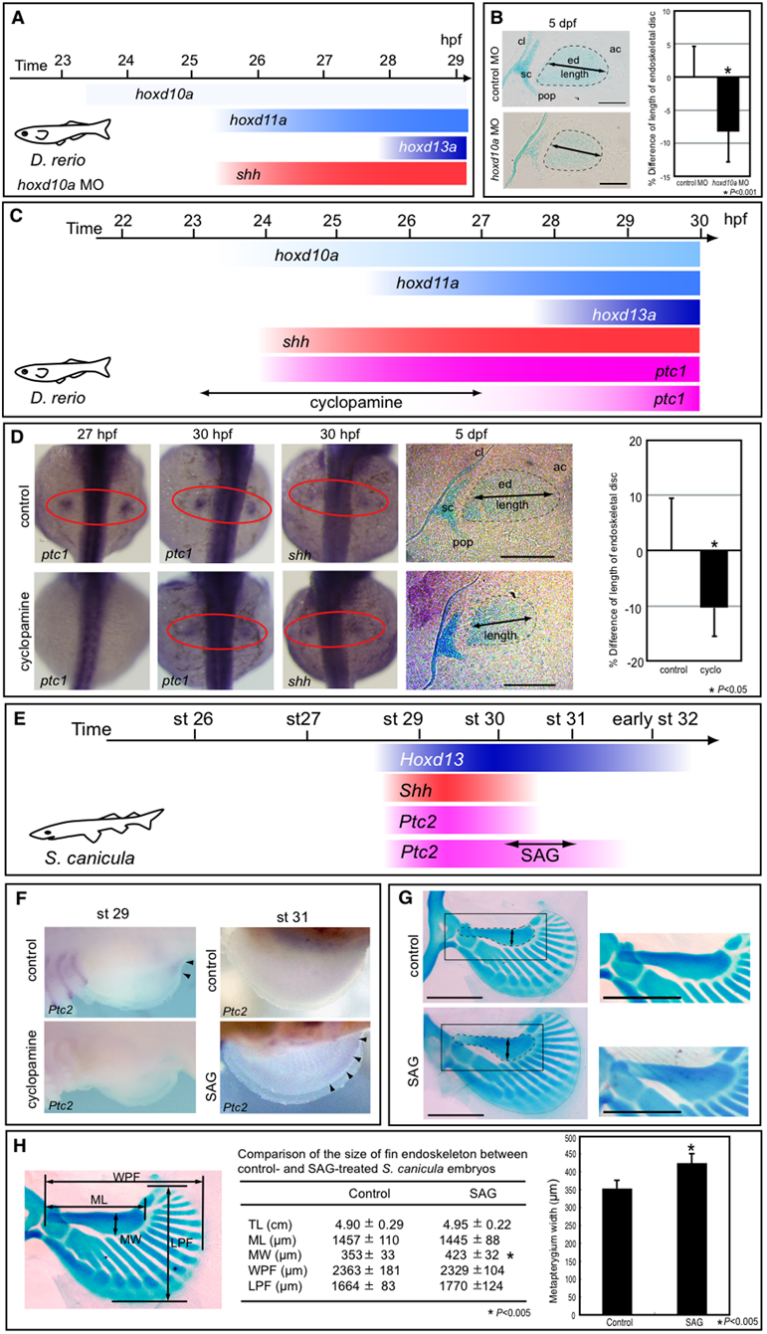
Temporal shift of Shh activity leads to changes in pectoral fin morphology. (A) *shh* expression appears at 25.5 hpf in pectoral fin primordia of *D. rerio* embryos injected with 5 ng of *hoxd10a* MO. (B) At 5 dpf, pectoral fins of embryos injected with control MO or with *hoxd10a* MO were stained with Alcian Blue (left). Cleithrum (cl), scapulocoracoid (sc), postcoracoid process (pop), endoskeletal disc (ed) and actinotrichs (ac) are indicated. Scale bars: 100 µm. The relative lengths of the endoskeletal disc are presented in the graph (right). *P<0.001, as assessed by Student's t-test. (C) *shh* expression appears at 24 hpf, concomitantly with *hoxd10a*, in pectoral fin primordia of *D. rerio*. Hedgehog signaling was blocked by treatment with 60 µM cyclopamine from 23 to 27 hpf, resulting in ablation of *ptc1* expression until at least 27 hpf. *ptc1* expression was recovered by 30 hpf in pectoral fin primordia of cyclopamine-treated embryos. (D) *ptc1* and *shh* expression were examined in control or cyclopamine-treated embryos at the indicated stages (left). At 5 dpf, pectoral fins of control or cyclopamine-treated embryos were stained with Alcian Blue (middle). Scale bars: 200 µm. The relative lengths of the endoskeletal disc are represented in a graph (right). *P<0.05, as assessed by Student's t-test with Welch's correction. (E) *Shh* and *Ptc2* expression disappeared before stage 31 in pectoral fin buds of *S. canicula* embryos. Hedgehog signaling was extended by treatment with SAG for 6 days from stage 30 to 31, resulting in extension of *Ptc2* expression until at least stage 31. (F) *Ptc2* expression was examined in control or SAG-treated embryos at stage 31 (5 days after the initial treatment). (G) Pectoral fins of control or SAG-treated embryos were stained with Alcian Blue. Anterior is to the left. Proximal is to the top. Insets show magnified views of the pectoral fin metapterygium. Note that the width of the metapterygium (arrows) of SAG-treated embryos was significantly increased. Scale bars: 1 mm. (H) Comparison of the size of the pectoral fin endoskeleton between control and SAG-treated *S. canicula* embryos. The table shows the total body length (TL), metapterygium length (ML), metapterygium width (MW), width across the base of pectoral fin endoskeleton (WPF), and length of pectoral fin endoskeleton (LPF) of control and SAG-treated embryos. The metapterygium lengths are represented in the bar graph. *P<0.05, as assessed by Student's t-test.

We next investigated whether a change in the timing of onset of *shh* expression induced by injection of *hoxd10a* MO could lead to a change in the zebrafish pectoral fin morphology (Fig. 5A and B). Zebrafish pectoral fins consist of an scapulocoracoid, a post-coracoid process, an endoskeletal disc, and actinotrichs at 5 days post-fertilization (dpf) [36]. Embryos were fixed and stained with Alcian Blue. Measurement of the endoskeletal discs of embryos injected with *hoxd10a* MO revealed that the total length of the disc along the proximal-distal axis was 8.41% shorter (*P*<0.001) compared with controls (control embryos, n = 8; *hoxd10a* MO injected embryos, n = 16; Fig. 5B).

To confirm that a change in the timing of Shh activity during fin development could modify fin size, we treated embryos between 23 and 27 hpf with 60 µM cyclopamine, a steroidal alkaloid that inhibits hh signal transduction (Fig. 5C and D). Control embryos showed expression of *ptc1*, a marker for the primary targets of hh signaling, in the posterior margin of fin primordia at 27 hpf and 30 hpf (n = 4 and 5, respectively, Fig. 5D). Expression of *ptc1* in cyclopamine-treated embryos was barely detectable in the fin primordia at 27 hpf (n = 5, Fig. 5D), whereas posterior activation of *ptc1* was readily detectable by 30 hpf (n = 9, Fig. 5D), indicating that cyclopamine treatment efficiently blocked hh signaling through 27 hpf. Thus, stimulation of an artificial heterochronic shift of Shh activity in the pectoral fin primordia was successful. Expression levels of *shh*, which are upregulated by a feedback loop of hh signaling, were normal in fin primordia of either ethanol- or cyclopamine-treated embryos at 30 hpf, indicating that Shh activity itself is not required for maintenance of *shh* expression between 23 and 30 hpf. Taken together, the results indicate that *shh* signal transduction was efficiently blocked in fin primordia of embryos treated with cyclopamine until 27 hpf, but signaling was recovered at least by 30 hpf. To examine the fin morphology at 5 dpf, embryos were fixed and stained with Alcian Blue. Measurements of the cyclopamine-treated endoskeletal discs revealed that the total length of the disc along the proximal-distal axis was 10.6% shorter than those of controls (ethanol-treated embryos, n = 7; cyclopamine-treated embryos, n = 9). The difference in the length between the ethanol- and cyclopamine-treated discs was significant at 0.05 levels by Student's t-test with Welch's correction (Fig. 5D). A longer exposure with cyclopamine until 57 hpf resulted in a more severe reduction (19.4%) in the length of the endoskeletal disc (ethanol-treated embryos, n = 7; cyclopamine-treated embryos, n = 9; Fig. S5). This reduction seemed to be depend on both the apical fold activity and shh activity [16]. These results indicate that the temporal shift of the onset of *shh* expression in pectoral fin primordia can lead to a change in the size of the endoskeletal discs along the proximal-distal axis in zebrafish embryos.

We then investigated whether hh signaling can be manipulated in pectoral fins of dogfish embryos (Fig. 5E–H). Prior to the treatment of dogfish embryos with SAG, agonists of smoothened [37], we tested whether SAG is applicable in live embryos using zebrafish and confirmed that we could manipulate hh activity by treatment with SAG (Fig. S5C). We then treated dogfish embryos with cyclopamine or SAG to test whether such treatment could modify hh signaling in developing dogfish embryos. At stage 29, *Ptc2* expression was observed in the posterior margin of pectoral fins of control embryos, whereas no *Ptc2* transcripts were detected in pectoral fins of cyclopamine-treated embryos (Fig. 5E). On the other hand, treatment with SAG resulted in extensive *Ptc2* expression in pectoral fins at stage 31 (Fig. 5E). These data demonstrated that hh signaling could be directly manipulated in dogfish embryos during fin development.

To examine whether the heterochronic shift of hh activity could alter the morphology of dogfish pectoral fins, we reared SAG-treated dogfish embryos for 11 to 12 weeks and then stained them with Alcian Blue. For SAG-treated embryos (n = 8), the width of the metapterygium was 19.8% greater compared with control embryos (n = 6; Fig. 5G, H). The difference in the metapterygium width between the control- and SAG-treated discs was significant by the Student's t-test with Welch's correction (*P*<0.005; Fig. 5H).

Taken together, our results indicate that altering the threshold levels of *hox* transcripts can trigger a heterochronic shift of *shh* expression in pectoral fin primordia, and the subsequent temporal shift of Shh activity causes changes in the size of the fin endoskeleton.

## Discussion

Our investigation of the genetic basis of vertebrate morphological evolution has yielded the following findings. (1) *Shh* expression appears as soon as there is a morphological bud in mouse and chick embryos (concomitant with *Hoxd10*), whereas *Shh* is transcribed very late (concomitant with *Hoxd13*) in pectoral fin buds of dogfish (*S. canicula*). (2) A threshold level of accumulated *hox* transcripts is critical for the timing of *shh* expression; specifically, if the amount of *hoxd10a* transcripts is below a threshold level, *shh* expression does not appear until *hoxd11a* is expressed in zebrafish. (3) A quantitative change of *hox* transcripts leads to changes in the size of the zebrafish endoskeleton. (4) A temporal shift in Shh activation in paired fins leads to a change in endoskeleton size in both dogfish and zebrafish.

### Heterochronic shift of *Shh* transcriptional onset depends on the quantity of *Hox*

Examination of collinear 5′-located *Hoxa* and *Hoxd* expression revealed that *Shh* expression was turned on when *Hoxd13* expression appeared, concomitant with a further increase in 5′-located *Hoxa* and *Hoxd* expression. These results raise the possibility that the late onset of *Shh* transcription in the pectoral fins of *S. canicula* embryos might correlate with either specific *Hox* transcripts or the overall expression level of *Hox* transcripts. Using zebrafish embryos, which allowed us to alter the levels of specific *hox* transcripts, we showed that the onset of *shh* expression is controlled by a certain threshold level of accumulated 5′-located *hox* transcripts. A recent study using *Hoxa*/*Hoxd* double mutant mice showed that there is a boundary between *Hoxd9*, the last *Hox* unable to elicit *Shh* transcription, and *Hoxd10*, the first *Hox* to activate *Shh* [3] —that is, between the genes expressed throughout the limb bud and those excluded from the anterior region. The authors proposed that the limb anterior-posterior polarity arises from the co-option of the collinear *Hox* gene expression across the main body axis [3]. Importantly, our experiments in dogfish showed that *Shh* transcripts do not appear until the onset of *Hoxd13* expression regardless of the nested posterior expression of *Hoxd10–12*. In other words, the *Shh* does not always initiate its expression even when the three penultimate *Hoxd* genes have already expressed posteriorly in paired appendages. The combination of experiments using both dogfish and zebrafish embryos has demonstrated that 5′-located *Hox* transcripts may not always reach the threshold levels required to stimulate *Shh* expression, even when the last four *Hox* paralog groups are expressed posteriorly. Absolute quantification of *Hox* gene transcripts necessary for *Shh* activation in mouse limb buds and in dogfish fin buds would allow us to further characterize the mechanisms by which *Hox* gene expression thresholds contribute to the evolution of vertebrate paired appendages. Although currently threre are no cartilaginous fishes amenable to transgenics manipulation or MO/mRNA injection, the prospective manipulation of *Hox* expression levels in these primitive gnathostomes should provide direct insight into our hypothesis of paired appendage evolution.

### *Hox* and co-factors in heterochronic shift of *Shh* activation

During vertebrate evolution, quantitative changes in *Hox* expression, Hox cofactors, and/or other unknown factors, could have shifted the onset of *Shh* expression, leading to changes in the morphology of endoskeleton. *Hox* genes act partially through the aid of co-factors, such as Meis and Pbx [38]. Although 5′-located *Hox* genes have been shown to act through *Meis*, we found that only *Pbx2* expression overlapped with *Shh* expression in pectoral fins in dogfish embryos (Fig. S6). Furthermore, manipulation of the level of *pbx2* expression in zebrafish embryos resulted in a change in the timing of the onset of *shh* expression in pectoral fin primordia in a low percentage of embryos (see Fig. S4 and S6). This may be due to a low level of *hox* in pectoral fin primordia. Alternatively, Pbx may make a smaller contribution than Hox to the activation of *Shh* expression. Biochemical approaches that address the roles of Hox co-factors in the onset of *Shh* expression will provide new insights into vertebrate limb evolution.

### Signalling pathways that control *Hox* expression levels

Signalling that regulates *Hox* transcriptional activation has been studied intensively. Retinoic acid is one of the factors thought to play key roles in controlling *Hox* gene transcription [39], [40], [41]. In zebrafish, a lack of retinoic acid in the pectoral fin buds results in the downregulation of *shh*, *hoxd11* and *hoxd12* [42]. In mice lacking retinoic acid-synthesizing enzyme gene–*retinaldehyde dehydrogenase 2* (*Raldh2*), *Shh* expression is greatly reduced in the limb buds and seen along the distal margin, whereas *Hoxd11* and *Hoxd12* are ectopically expressed in early limb buds [43]. *Hox* genes are differentially activated by retinoic acid in a concentration-dependent manner and in a sequential order that is collinear with their 3′ to 5′ arrangement in the cluster [44]. It would be interesting to explore whether retinoic acid reaches levels sufficient to activate 5′*Hoxd* genes at different times in the posterior paired appendages between dogfish and other tetrapods.

The zinc finger transcriptional factor GLI3 is another protein known to modulate *Hox* expression. In early limb buds of mouse embryos, GLI3 negatively regulates the expression of 5′-located *Hoxd* genes [45], [46]. In mouse and chick embryos, *Gli3* expression is excluded from the posterior part of the limb buds, when *Hand2* expression appears in the posterior region. *Gli3*, in turn, restricts *Hand2* expression in the posterior limb buds [47]. Such reciprocal antagonism seems to have been established in cartilaginous fishes, as *Hand2* expression is restricted to the posterior part of pectoral fins in *S. canicula* [20], indicating GLI3 may be involved in regulating *Hox* expression in the posterior region of dogfish fins. In addition, GLI3 physically interacts with HOXD12 during digit patterning [48]. In this regard, comparative analysis of the expression and function of *Gli3* with respect to *Hox* expression would enhance our understanding of the evolution of genetic networks involved in regulating *Shh* expression.

### Heterochronic shift of Shh onset in vertebrate fin evolution

**Figure 6:**
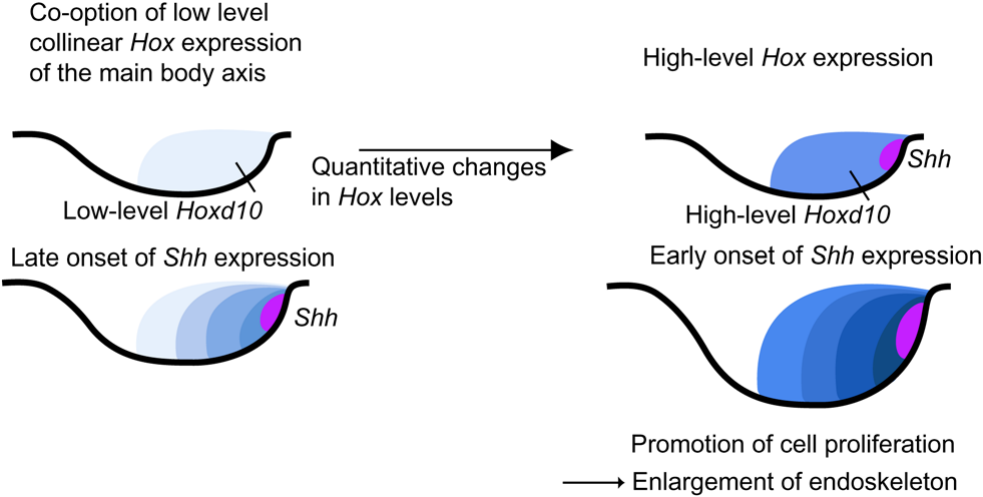
Diagram representing the effect of *Shh* expression heterochrony on vertebrate paired appendage evolution. A model suggesting that the early fin buds may have acquired low levels of *Hox* expression by co-option of collinear *Hox* expression in the main body axis [53]. Changes in accumulated *Hox* could have led to altered onset of *Shh* expression, resulting in enlargement of the endoskeletal elements during fin evolution.

Our results provide new clues for understanding the sequential events of vertebrate fin/limb evolution, especially with respect to the molecular mechanisms that change the onset of *shh* expression and lead to morphological changes in endoskeletal components (Fig. 6). It has been proposed that paired appendages adopted collinear expression of *Hox* from the main body axis concomitant with their emergence in the body wall [3], [49], [50] (Fig. 6). Our results suggest that if threshold levels of accumulated 5′ *Hox* transcripts were not reached, *Shh* expression may have been delayed or silent in ancestral fin buds. Quantitative changes in accumulated 5′ *Hox* may have led to altered onset of *Shh* expression, resulting in enlargement of endoskeletal elements during fin evolution (Fig. 6).

Endoskeletal components of paired appendages during the transformation from fins into limbs have been throughly discussed. Comparison of the paired appendages in fossils and in living primitive sarcopterygian fishes (lobe-finned fishes including lungfish and coelacanths) showed that endoskeletal elements of the paired appendages increased in size prior to the acquisition of the digital plates. Thus, the transition from fins to limbs seems to have required at least two major events, namely the enlargement of proximal endoskeletal elements with subsequent acquisition of digital plates. It has been proposed that the transformation of the apical fin fold into the short, apical ectodermal ridge may have promoted endoskeletal proliferation [4]. Here, we demonstrated that a temporal shift in Shh activity could have also led to changes in the size of the endoskeletal elements along the proximal-distal axis.

The effects of the late onset of *Shh* expression on limb morphology are difficult to examine using chick or mouse embryos because loss of Shh activity disrupts the Fgf/Shh positive feedback loop [51]. To circumvent this problem, we have taken advantage of the pectoral fin primordia of zebrafish embryos, in which *shh* expression occurs prior to the formation of the apical ectodermal ridge–like structure. We showed that temporal block of Shh signaling by cyclopamine, an inhibitor of hh signaling, prior to apical ridge formation can lead to a reduction in the size of fin endoskeletal elements (Fig. 5). Treatment with cyclopamine did not alter *shh* expression levels in fin primordia, indicating that Shh signaling recovers from cyclopamine treatment prior to the formation of the Fgf/Shh positive feedback loop [51]. Consistent with our proposal, studies in the zebrafish *sonic you* (*syu*) mutant, in which *shh* is disrupted, showed that shh in the early pectoral fin buds promotes cell proliferation that is at least partially independent of the apical fold, because a reduction in cell proliferation in *syu* fin buds was seen prior to the reduction of the apical fin fold and of *shh* expression [16]. In pectoral fin buds of the *syu* mutant, a more severe reduction in fin bud size was seen after ablation of the apical fold [16]. In tetrapod limbs, Shh together with Fgfs promote overproliferation of the posterior mesenchymal cells, leading to asymmetric growth of the limb [20], [51], [52]. Furthermore, recent studies revealed that Shh signalling controls not only the specification of digit progenitors but also cell proliferation in limb buds of chick embryos [14]. Thus, the temporal shift of *Shh* expression during vertebrate fin/limb evolution could have acted independently of, and/or synergistically with, Fgf signals from the apical fold, which also shift the timing of folding and promote cell proliferation, thereby contributing to the formation of the endoskeleton.

Because zebrafish larval pectoral fins are later remodeled to form the adult pectoral fins, it is difficult to speculate which endoskeletal components of paired fins among primitive fishes may have been affected by temporal changes in *shh* expression during evolution. Furthermore, the metapterygium was lost in the teleost lineage. Therefore, examination of these features in the paired appendages of the primitive cartilaginous dogfish is highly informative. Although dogfish embryos did not survive beyond 2 weeks after cyclopamine treatment (presumably due to the multiple malformations; data not shown), we have succeeded in keeping them alive for 12 weeks after treatment with SAG (Fig. 5G, H). We showed that extension of Shh activity using SAG could enlarge the metapterygium of dogfish pectoral fins. The metapterygium, a proximal component of the dogfish fin, has been considered to have persisted in sarcopterygian fishes and was the ancestral structure from which the tetrapod limb evolved. Enlargement of the dogfish fin metapterygium by extending Shh activity indicates that Shh could have promoted the proliferation of cells that formed the proximal structures among ancestral species. We propose that a heterochronic shift of the onset of *Shh* expression could have been mediated by changes in the level of Hox (and Hox co-factors) and that such transcriptional heterochrony could have influenced the proliferation of cells that contributed to the formation of endoskeletal components during vertebrate paired appendage evolution (Fig. 6). It would not be surprising if such a system controls the morphological diversification of paired appendages in different lineages (including lineages of cartilaginous fishes). It will be interesting to characterize these features of the body plan among different vertebrates having various types of paired appendages.

## Supporting Information

Figure S1 Expression of Shh and Fgfs during *S. canicula* fin development. (A) RT-PCR of *ScShh* in stage 29 and 32 *S. canicula* pectoral fin buds (left); results for stage 27 *S. canicula* embryos have been published [20]. The right panel shows semi-quantitative analysis of *ScShh* mRNA expression in pectoral fins relative to the *ScGAPDH* mRNA level. (B) Frontal view of the facial region at stage 27. (C–D, FG) Pectoral fin buds. Anterior is to the left. (B–D) *ScFgf8* expression at stage 27 (B, C) and 32 (D). Although transcripts were observed in nasal pits (np) and gill filaments (gf), no transcripts were detected in the apical fin fold (aff). (E) RT-PCR of *ScFgf8* in head (Head) and pectoral fins (Pec) of *S. canicula* embryos. (F) Staining of anti-Fgf4 antibody at stage 27. Arrowheads indicate anti-Fgf4-positive cells in the apical fin fold. (G) *Scβ-catenin* expression at stage 32. Abundant *Scβ-catenin* transcripts in pectoral fins including the apical fin fold (arrowheads) demonstrates probe efficacy.
(8.68 MB TIF)

Figure S2 Expression of *hoxd10a*, *hoxd11a* and *hoxd13a* during *D. rerio* pectoral fin development. Dorsal view of embryos injected with 5 ng of the control morpholino (MO) at 24, 25.5, 27 and 28 hpf. Red ovals highlight the pectoral fin primordia. Expression of *hoxd10a* was initially detected at 24 hpf, *hoxd11a* at 25.5 hpf, and *hoxd13a* at 28 hpf.
(8.08 MB TIF)

Figure S3 Quantitative PCR analyses of *hoxd11a* and *shh* expression in the lateral plate mesoderm of zebrafish embryos. Levels of *hoxd11a* (A) and *shh* (B–D) mRNAs in the lateral plate mesoderm of embryos were quantified relative to the *gapdh* mRNA level. (A–D) Expression levels of *hoxd11a* (A) and *shh* (B, C) in the lateral plate mesoderm of 24 hpf (B) and 25.5 hpf (A, C) embryos injected with 5 ng control, 1 ng *hoxd10a*, or 2.5 ng *hoxd10a* MO. (D) Quantitative PCR analyses to determine the expression levels of *shh* in the lateral plate mesoderm of embryos injected with 5 ng control MO, 5 pg *hoxd10a* mRNA, or 20 pg *hoxd10a* mRNA. Expression of *shh* was undetectable by quantitative PCR in 22.5 hpf injected with 5 ng control MO.
(2.32 MB TIF)

Figure S4 Onset of *shh* expression in zebrafish embryo fin primordia primarily depends on *hox* expression. The percentage of *D. rerio* embryos expressing the indicated level of *shh* transcript at 22.5, 24, or 25.5 hpf following injection of the indicated amount of control MO, *hoxd10a* MO, *hoxd13a* MO, *hoxd10a* mRNA, *hoxd13a* mRNA, *hoxa13a* mRNA, *pbx2* MO or *pbx2* mRNA is shown. A representative image depicting the detectable or undetectable levels of shh expression in the pectoral fin primordia is shown in Figure 2D.
(0.44 MB EPS)

Figure S5 Treatment of zebrafish embryos with cyclopamine or SAG. (A) Hedgehog signaling was blocked by treatment with 60 µM cyclopamine from 23 to 57 hpf, resulting in ablation of *ptc1* expression until at least 60 hpf. *ptc1* expression recovered by 72 hpf in pectoral fin primordia of cyclopamine-treated embryos. (B) *ptc1* expression was examined in control or cyclopamine-treated embryos at the indicated stages (left). At 5 dpf, pectoral fins of control (n = 7) or cyclopamine-treated embryos (n = 9) were stained with Alcian Blue (middle). The relative lengths of the endoskeletal disc are presented in the graph (right). *P<10^−6^, as assessed by Student's t-test. Cleithrum (cl), scapulocoracoid (sc), postcoracoid process (pop), endoskeletal disc (ed) and actinotrichs (ac) are indicated. Scale bars: 200 µm. (C) Zebrafish embryos were treated with SAG or cyclopamine, and *ptc1* expression was examined in adaxial cells. The specification of adaxial cells is known to depend on Hh signaling [1]. Panels show the dorsal view of *ptc1* expression in an 8-somite-stage control embryo (left), in a SAG-treated embryo (middle), and in a cyclopamine-treated embryo (right). In control embryo, adaxial cells are indicated by brackets. Note that *ptc1* expression is expanded in the SAG-treated embryo (brackets), whereas it is undetectable in the cyclopamine-treated embryo (right). 1. Wolff C, Roy S, Ingham PW (2003) Multiple muscle cell identities induced by distinct levels and timing of hedgehog activity in the zebrafish embryo. Curr Biol 13: 1169–1181.
(7.33 MB TIF)

Figure S6 The hox co-factor pbx makes a lesser contribution than hox to the onset of *shh* expression. (A) Expression of *Meis1* and *Pbx2* in the pectoral fin of *S. canicula* embryos at the indicated stages (top panels). Anterior is to the left. Arrowheads indicate transcripts in the proximal region. (B) Left: representative images depicting the detectability of *shh* expression in the pectoral fin primordia. Right: the percentage of *D. rerio* embryos with the indicated level of *shh* expression observed at 22.5, 24, and 25.5 hpf following injection of control MO, *pbx2* MO, or *pbx2* mRNA.
(9.69 MB TIF)
