# Supplementary figures and images for: Heterochronic Shift in Hox-Mediated Activation of Sonic hedgehog Leads to Morphological Changes during Fin Development
Source: PLoS One. 2009 Apr 13;4(4):e5121. doi: 10.1371/journal.pone.0005121 (PMC2664896; doi:10.1371/journal.pone.0005121)

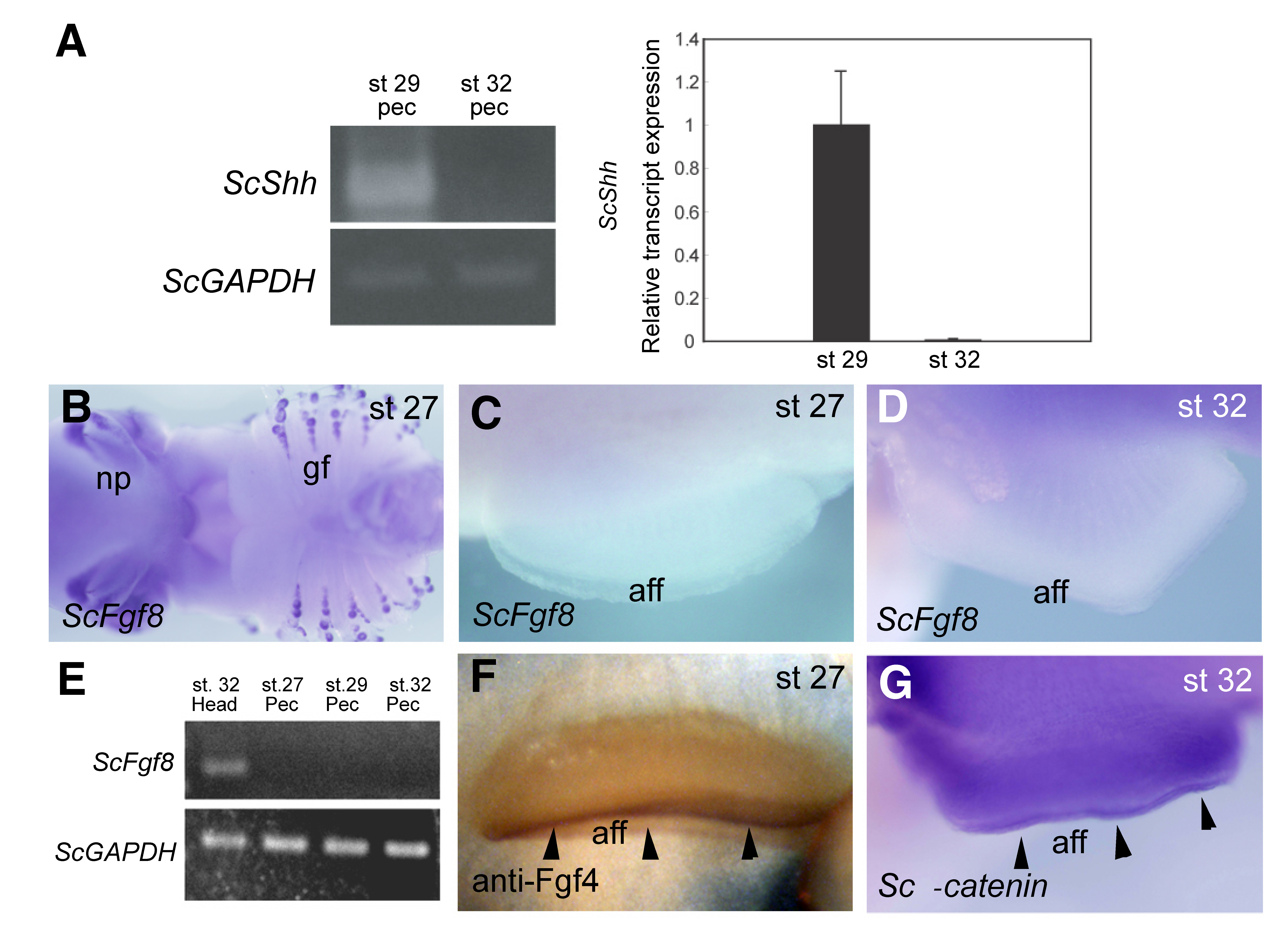

Supplement: Figure S1 — Expression of Shh and Fgfs during S. canicula fin development. (A) RT-PCR of ScShh in stage 29 and 32 S. canicula pectoral fin buds (left); results for stage 27 S. canicula embryos have been published [20]. The right panel shows semi-quantitative analysis of ScShh mRNA expression in pectoral fins relative to the ScGAPDH mRNA level. (B) Frontal view of the facial region at stage 27. (C–D, FG) Pectoral fin buds. Anterior is to the left. (B–D) ScFgf8 expression at stage 27 (B, C) and 32 (D). Although transcripts were observed in nasal pits (np) and gill filaments (gf), no transcripts were detected in the apical fin fold (aff). (E) RT-PCR of ScFgf8 in head (Head) and pectoral fins (Pec) of S. canicula embryos. (F) Staining of anti-Fgf4 antibody at stage 27. Arrowheads indicate anti-Fgf4-positive cells in the apical fin fold. (G) Scβ-catenin expression at stage 32. Abundant Scβ-catenin transcripts in pectoral fins including the apical fin fold (arrowheads) demonstrates probe efficacy. (8.68 MB TIF) [file pone.0005121.s001.tif]

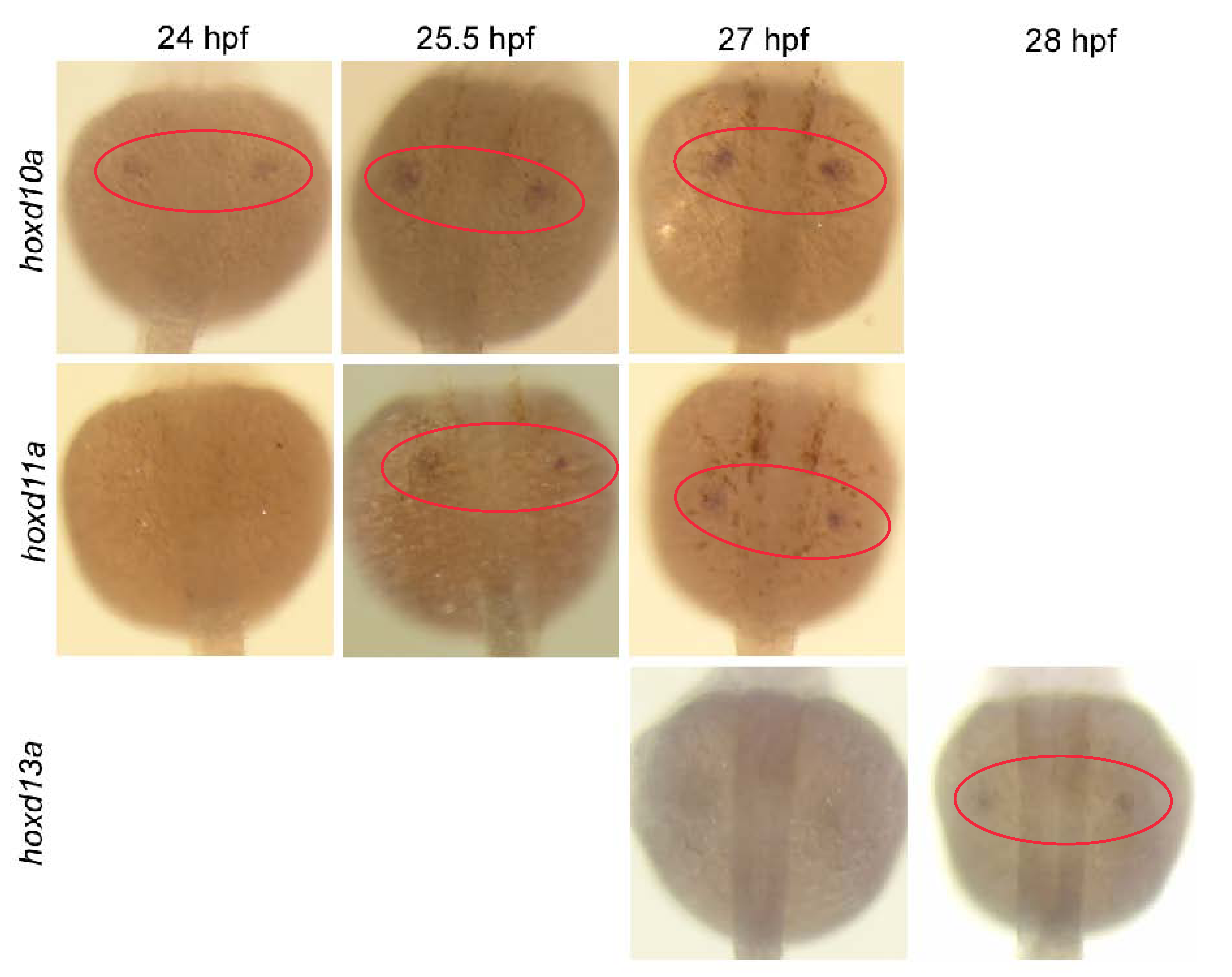

Supplement: Figure S2 — Expression of hoxd10a, hoxd11a and hoxd13a during D. rerio pectoral fin development. Dorsal view of embryos injected with 5 ng of the control morpholino (MO) at 24, 25.5, 27 and 28 hpf. Red ovals highlight the pectoral fin primordia. Expression of hoxd10a was initially detected at 24 hpf, hoxd11a at 25.5 hpf, and hoxd13a at 28 hpf. (8.08 MB TIF) [file pone.0005121.s002.tif]

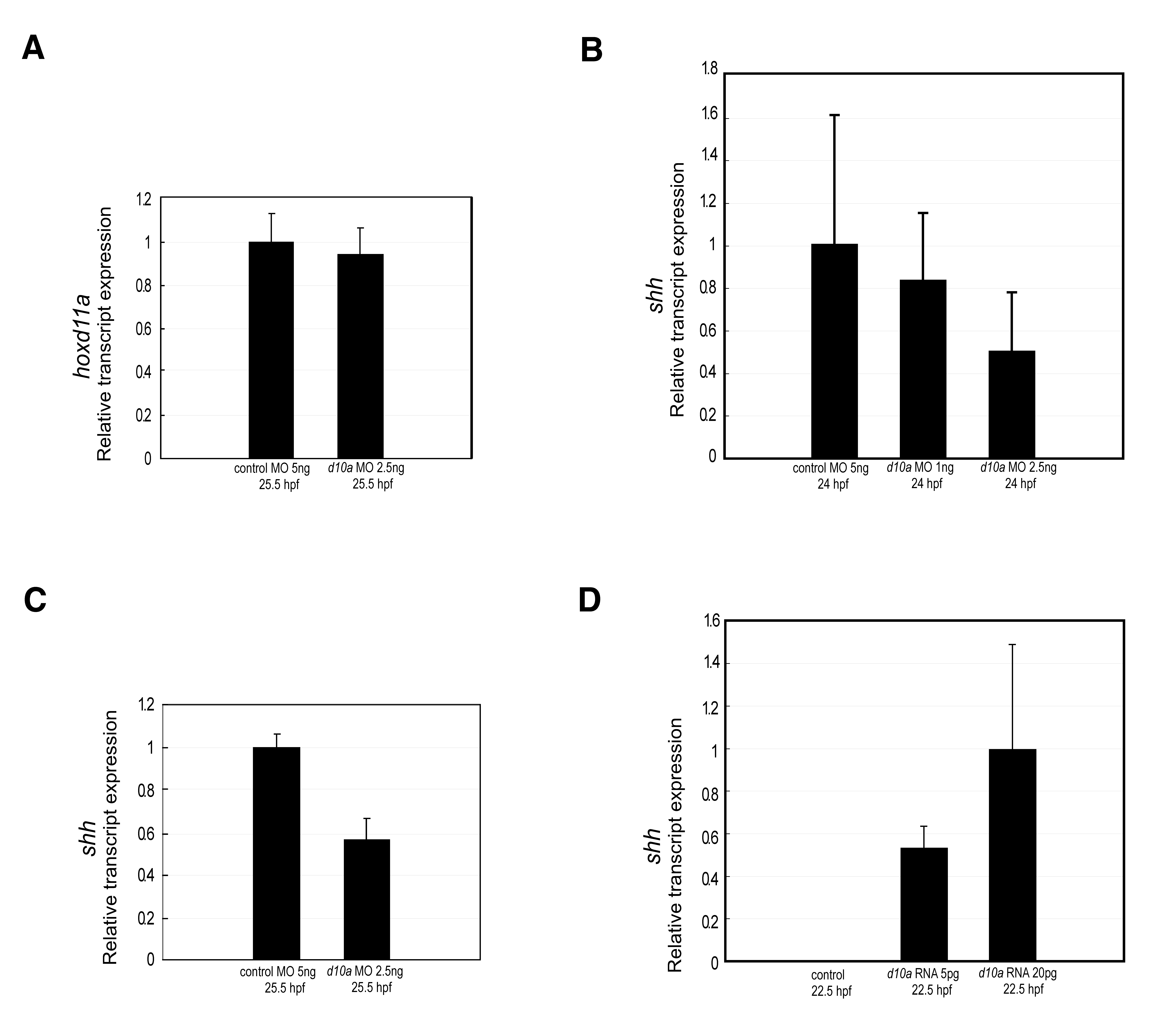

Supplement: Figure S3 — Quantitative PCR analyses of hoxd11a and shh expression in the lateral plate mesoderm of zebrafish embryos. Levels of hoxd11a (A) and shh (B–D) mRNAs in the lateral plate mesoderm of embryos were quantified relative to the gapdh mRNA level. (A–D) Expression levels of hoxd11a (A) and shh (B, C) in the lateral plate mesoderm of 24 hpf (B) and 25.5 hpf (A, C) embryos injected with 5 ng control, 1 ng hoxd10a, or 2.5 ng hoxd10a MO. (D) Quantitative PCR analyses to determine the expression levels of shh in the lateral plate mesoderm of embryos injected with 5 ng control MO, 5 pg hoxd10a mRNA, or 20 pg hoxd10a mRNA. Expression of shh was undetectable by quantitative PCR in 22.5 hpf injected with 5 ng control MO. (2.32 MB TIF) [file pone.0005121.s003.tif]

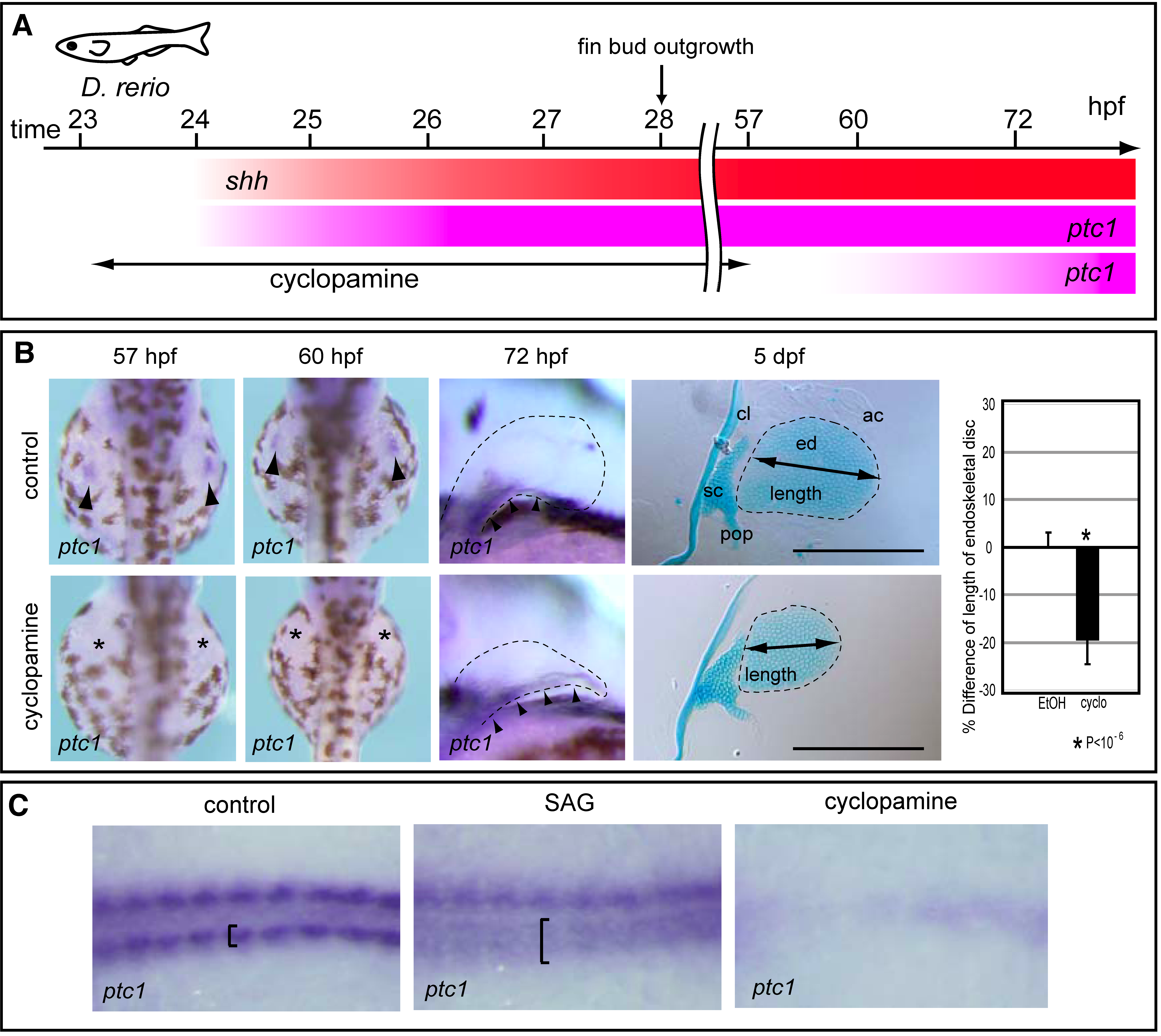

Supplement: Figure S5 — Treatment of zebrafish embryos with cyclopamine or SAG. (A) Hedgehog signaling was blocked by treatment with 60 µM cyclopamine from 23 to 57 hpf, resulting in ablation of ptc1 expression until at least 60 hpf. ptc1 expression recovered by 72 hpf in pectoral fin primordia of cyclopamine-treated embryos. (B) ptc1 expression was examined in control or cyclopamine-treated embryos at the indicated stages (left). At 5 dpf, pectoral fins of control (n = 7) or cyclopamine-treated embryos (n = 9) were stained with Alcian Blue (middle). The relative lengths of the endoskeletal disc are presented in the graph (right). *P<10−6, as assessed by Student's t-test. Cleithrum (cl), scapulocoracoid (sc), postcoracoid process (pop), endoskeletal disc (ed) and actinotrichs (ac) are indicated. Scale bars: 200 µm. (C) Zebrafish embryos were treated with SAG or cyclopamine, and ptc1 expression was examined in adaxial cells. The specification of adaxial cells is known to depend on Hh signaling [1]. Panels show the dorsal view of ptc1 expression in an 8-somite-stage control embryo (left), in a SAG-treated embryo (middle), and in a cyclopamine-treated embryo (right). In control embryo, adaxial cells are indicated by brackets. Note that ptc1 expression is expanded in the SAG-treated embryo (brackets), whereas it is undetectable in the cyclopamine-treated embryo (right). 1. Wolff C, Roy S, Ingham PW (2003) Multiple muscle cell identities induced by distinct levels and timing of hedgehog activity in the zebrafish embryo. Curr Biol 13: 1169–1181. (7.33 MB TIF) [file pone.0005121.s005.tif]
